# High-Throughput Sequencing of microRNAs in Glucocorticoid Sensitive Paediatric Inflammatory Bowel Disease Patients

**DOI:** 10.3390/ijms19051399

**Published:** 2018-05-08

**Authors:** Sara De Iudicibus, Marianna Lucafò, Nicola Vitulo, Stefano Martelossi, Rosanna Zimbello, Fabio De Pascale, Claudio Forcato, Samuele Naviglio, Alessia Di Silvestre, Marco Gerdol, Gabriele Stocco, Giorgio Valle, Alessandro Ventura, Matteo Bramuzzo, Giuliana Decorti

**Affiliations:** 1 Institute for Maternal and Child Health- IRCCS “Burlo Garofolo”, 34127 Trieste, Italy; sadeiu@libero.it (S.D.I.); stefano.martelossi@aulss2.veneto.it (S.M.); alessandro.ventura@burlo.trieste.it (A.V.); decorti@units.it (G.D.); 2 Department of Medicine, Surgery and Health Sciences, University of Trieste, 34127 Trieste, Italy; mlucafo@units.it; 3 Department of Biotechnology, University of Verona, 37100 Verona, Italy; nicola.vitulo@gmail.com; 4 CRIBI Biotechnology Centre, University of Padua, 35100 Padua, Italy; rosanna.zimbello@cribi.unipd.it (R.Z.); fabio.depascale@unipd.it (F.D.P.); claudio.forcato@gmail.com (C.F.); giorgio.valle@unipd.it (G.V.); 5 PhD School in Science of Reproduction and Development, University of Trieste, 34127 Trieste, Italy; samuele.naviglio@gmail.com (S.N.); alessia.disilvestre@phd.units.it (A.D.S.); 6 Department of Life Sciences, University of Trieste, 34127 Trieste, Italy; mgerdol@units.it (M.G.); stoccog@units.it (G.S.)

**Keywords:** glucocorticoids, mRNA, inflammatory bowel disease, pediatric patients

## Abstract

The aim of this research was the identification of novel pharmacogenomic biomarkers for better understanding the complex gene regulation mechanisms underpinning glucocorticoid (GC) action in paediatric inflammatory bowel disease (IBD). This goal was achieved by evaluating high-throughput microRNA (miRNA) profiles during GC treatment, integrated with the assessment of expression changes in GC receptor (GR) heterocomplex genes. Furthermore, we tested the hypothesis that differentially expressed miRNAs could be directly regulated by GCs through investigating the presence of GC responsive elements (GREs) in their gene promoters. Ten IBD paediatric patients responding to GCs were enrolled. Peripheral blood was obtained at diagnosis (T0) and after four weeks of steroid treatment (T4). MicroRNA profiles were analyzed using next generation sequencing, and selected significantly differentially expressed miRNAs were validated by quantitative reverse transcription-polymerase chain reaction. In detail, 18 miRNAs were differentially expressed from T0 to T4, 16 of which were upregulated and 2 of which were downregulated. Out of these, three miRNAs (miR-144, miR-142, and miR-96) could putatively recognize the 3’UTR of the GR gene and three miRNAs (miR-363, miR-96, miR-142) contained GREs sequences, thereby potentially enabling direct regulation by the GR. In conclusion, we identified miRNAs differently expressed during GC treatment and miRNAs which could be directly regulated by GCs in blood cells of young IBD patients. These results could represent a first step towards their translation as pharmacogenomic biomarkers.

## 1. Introduction

Paediatric onset inflammatory bowel disease (IBD) is generally more extended, more severe, and progresses more rapidly than the form occurring in adulthood. In spite of the introduction of highly effective biological agents in the therapy of this disease, glucocorticoids (GCs) are still employed to induce remission in moderate to severe forms, especially in ulcerative colitis. Nevertheless, considerable inter-individual differences in efficacy and side effects have been reported. Although several studies have increased the knowledge about the mechanism of action of GCs over the past few years [1], research on the personalization of GC therapy by pharmacogenetics in IBD has only achieved modest success to date. A potential genetic marker is the glucocorticoid receptor gene (*NR3C1*), located in chromosome 5: after GC binding, the glucocorticoid receptor (GR) translocates into the nucleus and binds palindromic DNA-binding sites, the so-called glucocorticoid responsive elements (GREs), localized in the promoter region of target genes [2,3,4]. Upon DNA binding, GCs can induce trans-activation and trans-repression processes, thus controlling gene expression. GR activity is conditioned by the chaperone proteins heat shock protein 70 and 90, and by a number of co-chaperones, including STIP1 and the immunophilins FKBP4 and FKBP5, which altogether form a molecular heterocomplex with GR itself, that is required for proper ligand binding, receptor activation, and transcription [2]. Abnormalities in proteins involved in the formation of the heterocomplex may contribute to altered GC responsiveness [3]. Although several studies have demonstrated that components of the heterocomplex show altered gene expression profiles in the comparison between steroid resistant and responder patients, it is still unclear whether these changes are caused by the variability in individual response or are the consequence of GC treatment [4,5,6].

These observations pinpoint the need to carry out further studies aimed to investigate the complex gene regulatory network mediated by GCs. Within this context, non-coding microRNAs (miRNAs) have emerged as important gene expression regulatory elements.

MicroRNAs are small (18–24 nucleotides) non-coding RNAs, which bind the 3′UTRs of their target genes and inhibit their expression [7], either by mediating messenger RNA (mRNA) cleavage (most common in plants) or by translational repression (most common in metazoans) [8,9]. A single miRNA can regulate approximately 200 mRNAs, and each mRNA can in turn be regulated by multiple miRNAs [7]. Emerging data have implicated the deregulated expression of certain miRNA networks in the pathogenesis of autoimmune and inflammatory diseases, including IBD, and they have been suggested to play an important role in these diseases [8,9,10,11,12].

In spite of the growing interest in identifying the role of miRNAs as modulators of genes involved in drug response, the number of studies on this topic is still very limited, to the point that no data is presently available about miRNA regulation by GCs in IBD, particularly in children. The aim of this study was to obtain high-throughput miRNA profiles in paediatric IBD patients at diagnosis and after the first cycle of GC therapy, used to induce remission, in order to identify miRNAs significantly deregulated by steroid treatment. In parallel, using a candidate gene approach aimed at evaluating selected genes involved in the molecular mechanism of response to GC treatment, we assessed the expression changes of GR (gene name *NR3C1*), heterocomplex genes (*FKBP4*, *FKBP5*, and *STIP1*), and GILZ (gene name *TSC22D3*, upregulated by GCs). The integration of these results might provide important information about how miRNAs regulate the selected genes involved in the mechanism of action of GCs. Finally, we investigated whether GCs could directly regulate miRNAs containing GRE sequences in their gene promoter regions.

## 2. Results

### 2.1. miRNA Analysis

Ten paediatric IBD patients were enrolled at diagnosis at the Paediatric Clinic of IRCCS Burlo Garofolo in Trieste in a prospective study. These patients (mean age at enrolment 12.6 years, range 6.2–17.8 years; 8 ulcerative colitis and 2 Crohn’s disease; 5 males and 5 females) were treated with prednisone 1 to 2 mg/kg/day for 4 weeks.

**Figure 1:**
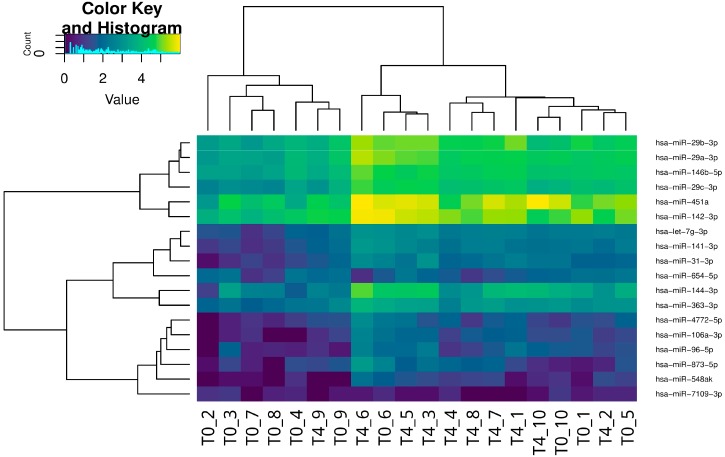
Heatmap of the normalized read counts which represent the hierarchical clustering of miRNA expression in each patient at the onset of the disease (T0) and after four weeks of steroid treatment (T4).

Using the procedure described in Materials and Methods, we were able to align on a mirBase miRNA dataset an average of 4 million reads for each sample (project accession number PRJNA297769). The differential expression analysis identified 18 miRNAs that were differentially expressed from T0 to T4 and potentially regulated by GCs (Figure 1). In detail, a total of 16 miRNAs were upregulated and 2 downregulated. The absolute fold changes ranged from 2.21 to 4.44 for up-regulated miRNAs, and from −4.62 to −2.27 for the downregulated ones. Table 1 reports the list of GC-sensitive miRNAs; those previously linked in the literature with GC regulation or whose target genes are known to be under GC control are indicated.

**Figure 2:**
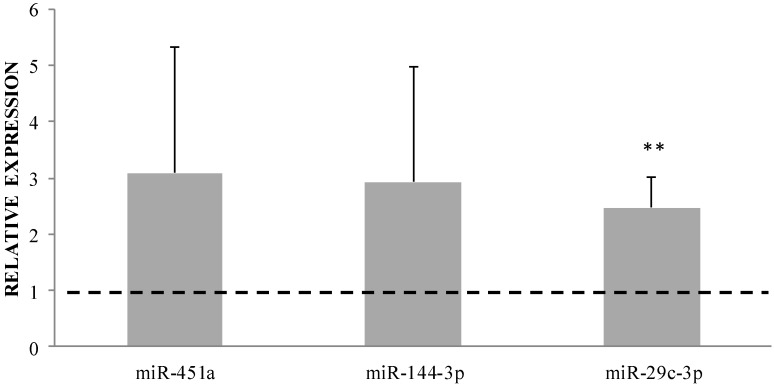
Relative expression of miR-451a, miR-144-3p, and miR-29c-3p (calculated as 2^−ΔΔ*C*t^ T4 vs. T0). Values > 1 (dotted line) indicate upregulation, values < 1 indicate downregulation. Parametric *t*-test Δ*C*_t_ T0 vs. T4, ** *p* < 0.01.

Three up-regulated miRNAs (i.e., miR-451a, miR-144-3p, and miR-29c-3p) were selected to validate these results based on the magnitude of regulation and previous knowledge associating these miRNAs to GC effects. A good agreement was observed between miRNA-seq and qRT-PCR validation results (Figure 2).

Three out of the 18 GC-regulated miRNA genes contained positive GC responsive element sequences (pGREs) in their promoter regions, which represents the site potentially responsible for a direct regulation by the GC receptor (Table 2). In detail, all three miRNA genes (i.e., miR-363, miR-96, and miR-142) were upregulated.

### 2.2. mRNA Analysis

Candidate gene expression evaluations were performed using Taqman gene expression assays: the regulation from T0 to T4 of GR heterocomplex genes (*NR3C1*, *FKBP4*, *FKBP5*, and *STIP1*) and GILZ, a gene upregulated by GCs, were evaluated.

**Figure 3:**
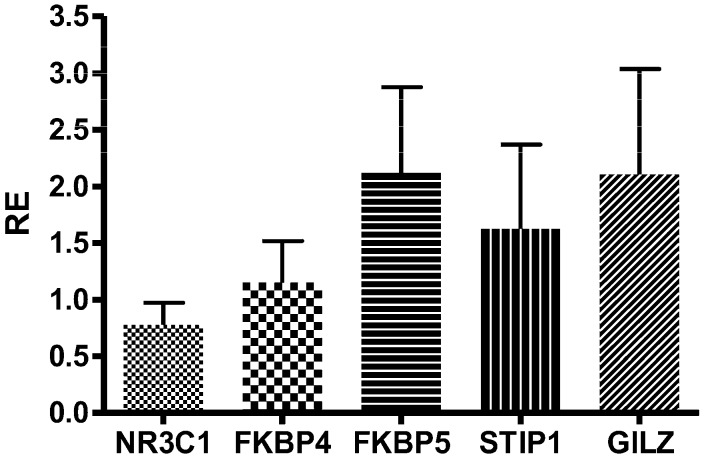
Relative expression (RE) of NR3C1, FKBP4, FKBP5, STIP1, and GILZ (calculated as 2^−ΔΔ*C*t^ T4 vs. T0). Parametric *t*-test Δ*C*_t_ T0 vs T4.

The expression of these genes did not significantly change after GC treatment (GILZ *p* = 0.99, CI −1.351 to 1.361, mean RE = 2.10 ± 2.94; FKBP4 *p* = 0.28, CI −1.862 to 0.614, mean RE = 1.15 ± 1.16; FKBP5 *p* = 0.86, CI −1.781 to 1.515, mean RE = 2.12 ± 2.39; STIP1 *p* = 0.44, CI −1.989 to 0.937, mean RE = 1.62 ± 2.35). Only for NR3C1 was a trend of reduction observed (*p* = 0.07, CI −1.943 to 0.111, median RE = 0.77 ± 0.62; Figure 3).

### 2.3. Integration between miRNA and mRNA Analysis

The database miRtarBase was used to identify mRNAs potentially targeted by miRNAs deregulated by GCs. Among the 18 GC sensitive miRNAs, three (i.e., miR-144-3p, miR-142-3p, and miR-96-5p) could putatively recognize (with weak evidence validation methods) the 3’UTR of the GR gene. In particular, miR-144-3p, the most upregulated among GC-sensitive miRNAs in our patients, miR-142-3p and miR-96-5p, can potentially recognize the 3′-UTR of the NR3C1 mRNA, which showed a decreased expression after GC treatment [14,20]. Our analyses could not identify any miRNA that could recognize the 3′-UTR of FKBP4, FKBP5, STIP1, and GILZ mRNA based on mirTarBase database information.

## 3. Discussion

The most important aim of our research was the identification of miRNAs modulated by GC treatment.

Among all human miRNAs analyzed, 18 were differentially expressed from T0 to T4. In detail, 16 miRNAs were upregulated and two were downregulated. Although our analysis could not enable a precise discrimination between a direct effect of GCs or the consequences of resolved inflammation, some of the miRNAs identified as deregulated after treatment in our patients have previously shown a similar trend in different cellular models treated with GCs [13,15], leading us to hypothesize that their alteration could depend on GCs themselves. Interestingly, the results of other studies aimed at the identification of differentially expressed miRNAs in IBD patients treated with other drugs, such as infliximab [21], found no overlap with miRNAs identified in our study, suggesting that the alteration we observed is likely dependent on GC treatment.

It is important to point out that this is the first report about the evaluation of miRNA regulation in cells of IBD patients treated with GCs for the induction of remission, and that only a single paper has been recently published on the role of differentially expressed serum miRNAs in children affected by IBD after treatment with prednisone [22]. We selected GC sensitive patient cells to obtain a good pharmacological model to better understand the complex mechanism of GC gene regulation in which miRNAs could be involved.

Among the 18 GC-sensitive miRNAs, we could identify three (i.e., miR-144-3p, miR-142-3p, and miR-96-5p) which can putatively recognize the 3′UTR of the GR mRNA. In particular, miR-144-3p, which was the most upregulated among GC-sensitive miRNAs in our patients, was reported to positively affect the expression of GRβ in T24 human bladder cancer cells [23]. No in vitro data are available for miR-144-3p and human GR, but bioinformatic investigations conducted by Hafner and colleagues [14] revealed that this miRNA targets sites on the GR 3′UTR region of human embryonic kidney 293 cells. Data on miR-96-5p and miR-142-3p published by Riester and colleagues [14] determined the miRNA expression pattern in mouse adrenal glands under baseline conditions, as well as in response to Adreno Cortico Tropic Hormone (ACTH) stimulation. The authors found that, when miR-96 or miR-142-3p were cotransfected with a vector carrying the 3′UTR region of the NR3C1 gene into HEK293T cells, the expression of 3′UTR was significantly down-regulated, as predicted in silico. The increased expression of these three miRNAs observed in our study after GC treatment could be responsible for the decreased expression of NR3C1 observed at T4 in our patients. Further studies should evaluate by flow cytometry whether the level of expression of GR in peripheral blood mononuclear cells (PBMCs) decreases after treatment.

FKBP5, a member of the GR heterocomplex, was upregulated after GC treatment in our patients (mean Fold Changes: 2.12 ± 2.39). This gene contains GREs which can be directly bound by GR [24]. Moreover, GR sensitivity is additionally regulated by GCs through the induction of FKBP5 expression, thereby establishing an intracellular ultra-short negative feedback loop [25], which might sustain the upregulation that was observed in our analysis.

Expression of miRNAs is controlled by the same mechanisms of protein coding genes, including upstream regulatory elements (i.e., promoters). In this context, one of the most important findings of our research was the characterization of the presence of GREs in the promoter of some of the GC-sensitive miRNA genes identified. The consensus pGRE is composed of two hexamer half sites separated by three random nucleotides. A majority of GREs contain the hexamer half-site sequence TGTTCT. The number and position of GREs within gene promoter regions influences the intensity of the transcriptional response [26].

A total of three miRNAs upregulated by GCs contained GREs. Consistently with their expression trends, these miRNA genes presented pGREs. We can hypothesize that GCs transcriptionally regulate the expression of some miRNAs through a GC receptor-mediated direct DNA-binding mechanism. To the best of our knowledge, GRE motif analysis has never been carried out on miRNAs regulated by GCs in our study. The only exception is represented by a single paper by Kong and colleagues [27], which identified a GRE sequence in the promoter of the miR-27b gene, found to be upregulated by dexamethasone. Out of the three miRNA genes containing GREs, miR-96 and miR-142 have been previously related to regulation by GCs.

In particular, miR-96 modulates the transcription factor FoxO1/3, involved in GC-induced apoptosis [13], and as discussed above, both miR-96 and miR-142 could play a role in the regulation of GR expression [14].

Additional experiments will be required to address the significance of these findings, the binding characteristics of the identified miRNAs, and their functional activity after exposure to GCs. Moreover, it would be interesting to compare differential miRNA expression in cases where remission is induced by a different therapy, to control for confounding effects independent from GCs.

In conclusion, the expression of 18 miRNAs was modulated after GC treatment in IBD paediatric patients. Three of these miRNAs presented GREs, suggesting that GCs may possibly transcriptionally regulate their expressions through a GC receptor-mediated direct DNA-binding mechanism. Moreover, the changes observed in the expression of the GR gene, occurring during GC therapy, could be to some extent linked to the regulation of miRNA by GCs. Although these results need to be confirmed and validated in vitro, they provide new information on the possible molecular mechanism of action of GCs and the consequent transcriptional regulation.

This study evaluated differently expressed miRNAs during GC treatment in blood cells (PBMC) of young IBD patients enrolled at diagnosis and followed for the first four weeks of steroid therapy. Even though a mixed cells analysis is suboptimal, since differences in gene expression can be driven by differences in the cellular composition of starting material, the investigation of miRNA expression in PBMCs represents a simple approach with diagnostic purpose, and easier to translate in clinical practice. Moreover, we decided to focus our analyses on the cellular targets of GCs (PBMCs), hypothesizing a stronger effect of GC treatment on miRNA expression in this tissue, compared to that observable in whole blood, plasma or serum, which may be easier to collect, but may also provide expression profiles somewhat confounded by miRNAs originating from tissues not directly involved in GC effects.

While the number of patients studied is small, this project design, which includes patients evaluated at diagnosis and drug naïve, can reduce to the minimum the effect of confounding factors, and should therefore support findings with greater statistical confidence. This report may represent a first important step towards further studies on relevant candidate GC-sensitive miRNAs.

Future investigations should be addressed at confirming these results in a larger number of paediatric patients affected by IBD, and divided on the basis of their clinical response to GCs. In detail, differences in the expression of miRNAs and mRNAs eventually observed at diagnosis between GC responders and non-responders, as well as variations upon GC treatment, should be investigated to obtain pharmacogenomic biomarkers useful to predict GC response in paediatric IBD patients. Furthermore, the identification of pharmacological and molecular determinants associated with GC response in paediatric IBD patients may potentially improve their treatment. Personalization of therapy based on this information will result in higher quality of life, lower toxicity, and a more rational treatment.

## 4. Materials and Methods

### 4.1. Patients

Ten paediatric IBD patients were enrolled at diagnosis at the Paediatric Clinic of IRCCS Burlo Garofolo in Trieste in a prospective study. These patients (mean age at enrolment 12.6 years, range 6.2–17.8 years; 8 ulcerative colitis and 2 Crohn’s disease; 5 males and 5 females) were treated with prednisone 1 to 2 mg/kg/day for 4 weeks. Peripheral blood was collected from enrolled patients at diagnosis (T0) and after 4 weeks of prednisone treatment (T4). Clinical activity, inclusive of clinical and inflammatory markers evaluation, was assessed using the “Pediatric Crohn’s Disease Activity Index” (PCDAI) for patients with Crohn’s Disease, and with the “Pediatric Ulcerative Colitis Activity Index” (PUCAI) for patients with ulcerative colitis. Clinical remission was defined as PCDAI < 10 or PUCAI < 10, and clinical improvement was defined as a reduction of at least 15 points from baseline score. These evaluations were performed at the diagnosis (before treatment), and after 4 weeks of treatment with prednisone. All patients showed an improvement of symptoms after GC therapy at week 4 with PCDAI = 5 and PUCAI = 3.8. These patients were hence considered GC responders and evaluated for genomic and bioinformatic analysis.

### 4.2. RNA Extraction

The PBMCs of patients were collected by density gradient centrifugation on Ficoll PaqueTM Plus (Healthcare, Milan, Italy) at diagnosis and after 30 days of therapy. The PBMCs were preserved in RNAlater (Thermo Scientific, Waltham, MA, USA) at −80 °C up to the extraction, which was done with PureLink RNA Mini kit (Thermo Scientific, Waltham, MA, USA), according to the manufacturer’s protocol.

The RNA concentration and purity were calculated by Nano Drop instrument (NanoDrop 2000, Thermo Scientific, Waltham, MA, USA). The amount and quality of small RNAs in the total RNA sample was first determined with both the Agilent RNA 6000 NanoKit (Agilent, Santa Clara, CA, USA) to generate a RIN score and the Agilent Small RNA Kit to calculate the concentration of miRNAs in the total RNA on an Agilent 2100 Bioanalyzer.

### 4.3. miRNA-seq

The amount of miRNA used in the following steps was targeted at 1–25 ng, maintaining the total RNA amount to less than 1 μg in a volume of 3 μL. The libraries were prepared with the Ion Total RNa-seq Kit (Life Technologies, Carlsbad, CA, USA), according to manufacturer’s instructions, and the final amplified product size was about 105 bp. The sequencing reaction was performed on an Ion Proton platform (Life Technologies, Carlsbad, CA, USA) setting 160 flows and the mean fragment size was about 20–25 bp.

### 4.4. miRNAs Identification

Ion Proton reads were pre-processed using the cutadapt software [28] to trim the adaptor sequence (ATCACCGACTGCCCATAGAGAGGCTGAGAC) and to remove bases with a quality score lower than 17. All the reads with a final length lower than 15 bases were removed and were not considered for further analysis. The remaining reads were aligned against the *H. sapiens* miRBase hairpin database using PASS aligner. The reads were mapped using the local alignment mode and allowing one mismatch. MicroRNAs were quantified considering only those reads that completely covered a mature miRNA. Moreover, reads finding multiple matches (i.e., those mapping on multiple gene loci) were evenly distributed among matching genes.

### 4.5. Differential miRNA Expression Analysis

The differential miRNA expression analysis between the samples collected at diagnosis and after 4 weeks of steroid treatment was performed using the edgeR package [29]. To take into account the different sequencing depth among the samples, the read counts were normalized using the full quantile normalization method implemented in the EDASeq package [30]. Differentially expressed miRNAs were identified based on a FC threshold of |2|, coupled with a False Discovery Rate-corrected *p*-value lower than 0.05.

### 4.6. Validation of Selected Differentially Expressed miRNAs by qRT-PCR

From the list of the top differentially expressed miRNAs, three up-regulated miRNAs (i.e., miR-451a, miR-144-3p, miR-29c-3p) were selected for their expression validation. The expression of these miRNAs for the validation panel was measured by qRT-PCR TaqMan^®^ analysis using the CFX96 real-time system-C1000 Thermal Cycler (Bio-Rad Laboratories, Hercules, CA, USA). First, each miRNA was specifically reverse-transcribed to cDNA using TaqMan miRNA RT-Kit with stem-loop RT-primer (Applied Biosystem, Foster City, CA, USA), according to the manufacturer’s protocol. Second, PCR products were amplified from cDNA samples using the TaqMan MicroRNA Assay together with the TaqMan^®^ Universal PCR Master Mix (Applied Biosystem, Foster City, CA, USA). RNU48 was used as a reference gene for internal normalization. The relative expression levels of miRNAs before (T0) and after treatment with GCs (T4) were calculated using the comparative Ct method (2^−ΔΔ*C*t^ method).

### 4.7. TaqMan Gene Expression Analysis

Expression levels of the selected candidate genes (*NR3C1*, *FKBP4*, *FKBP5*, *STIP1*, and *TSC22D3*), were evaluated by qRT-PCR TaqMan^®^ analysis using the CFX96 real-time system-C1000 Thermal Cycler (Bio-Rad Laboratories, Hercules, CA, USA). The reverse transcription reaction was carried out with the High Capacity RNA-to-cDNA Kit (Applied Biosystem, Foster City, CA, USA) and the qRT-PCR was performed in triplicate using the TaqMan^®^ Gene Expression Assay according to the manufacturer’s instructions. The thermal cycling conditions for TaqMan assays were as follows: 2 min at 50 °C and 10 min at 95 °C, followed by 40 cycles at 95 °C for 15 s and 60 °C for 60 s. The expression levels were evaluated using the comparative *C*_t_ method (2^−ΔΔ*C*t^ method). *C*_t_ values were corrected based on PCR efficiencies using LinRegPCR. Results were normalized using the 18S as a housekeeping gene and reported as Relative Expression (RE) value.

### 4.8. Identification of Putative mRNA Targets

The list of mRNA targets was downloaded from mirTarBase [31], which contains experimentally validated microRNA-target interactions. Depending on the type of experiment, the evidence is annotated as strong (Reporter assay, Western blot, and qPCR) or weak (Microarray, NGS, pSILAC).

### 4.9. Identification of GC Responsive Elements (GRE)

The positive GC Responsive Elements (pGRE) were identified using FIMO (Find Individual Motif Occurrences) [32], part of the MEME suite [33]. We used the online web tool available at http://meme-suite.org/tools/fimo. The positional weight matrix of the GRE motif was downloaded from TRASFAC database, release 7.0 (accession code: M00205) [34], and was searched in the 2 kb regions upstream of each differentially expressed miRNA gene. To discriminate between true motif and background noise, the significance threshold was set to a *p* value ≤ 0.0001.

The identification of the negative GREs (nGRE) was performed using the sequences proposed by Surjit et al. [35]. The sequences were searched using a home-made Perl script to scan the promoter regions of miRNA with the following regular expression CTCC[A,C,G,T]0,2GGAGA and its reverse complement in case the miRNA was coded in the reverse strand.

### 4.10. Statistical Analysis

Statistical analyses were performed using R software (ver. 2.9.1). Differential expression between T0 and T4 in each gene was investigated by calculating an odds ratio (OR) and 95% confidence intervals (CI) from contingency tables, and using paired *t*-test. The threshold level of statistical significance was set to ≤0.05 after Benjamini–Hochberg FDR correction.

### 4.11. Ethical Considerations

Local ethical committee (Comitato indipendente per la bioetica, Istituto di Ricovero e Cura a Carattere Scientifico materno infantile Burlo Garofolo, Trieste, Italy) approval for the study (Prot 2198; approval date: 17 September 2013) was provided. All patients participated in this study in accordance with the principles outlined in the Declaration of Helsinki, and written informed consent was obtained from each participating patient and/or their parents or guardians.

## Figures and Tables

**Table 1:** Differentially expressed miRNAs.

Upregulated miRNAs	FC	FDR Corrected *P*-Value	Downregulated miRNAs	FC	FDR Corrected *p*-Value
hsa-miR-451a * [13] hsa-miR-144-3p * [14,15] hsa-miR-96-5p * [13,14] hsa-miR-29b-3p * [13] hsa-miR-142-3p * [14] hsa-miR-873-5p hsa-miR-29c-3p * [16,17] hsa-miR-29a-3p * [13] hsa-miR-363-3p hsa-miR-141-3p hsa-miR-548ak hsa-let-7g-3p* [18] hsa-miR-4772-5p hsa-miR-106a-3p hsa-miR-31-3p hsa-miR-146b-5p * [19]	4.16 4.44 2.96 2.89 2.21 3.36 3.37 2.72 2.31 2.59 3.11 2.44 2.70 3.52 3.36 2.27	1.66 × 10^−6^ 1.04 × 10^−5^ 6.38 × 10^−3^ 0.026 0.026 0.026 0.037 0.041 0.041 0.041 0.042 0.042 0.047 0.047 0.049 0.049	hsa-miR-7109-3p hsa-miR-654-5p	−4.62 −2.27	0.044 0.049

Fold changes (FC) for each miRNA regulated by glucocorticoids (GCs); * Linked to GC regulation in the literature. FDR, False Discovery Rate.

**Table 2:** Glucocorticoid responsive element (GRE) sites predicted on miRNA promoter regions.

miRNA	pGRE	Start	End	Strand	Chrom	Expression
hsa-miR-363	GTGATAATGTGTGCTT	133303695	133303710	−	chrX	Up
hsa-miR-96	AGGACAAAGAGTCCTC	129416083	129416098	−	chr7	Up
hsa-miR-142	CTCACCTTCAGTTCTG	58331606	58331621	+	Chr17	Up
hsa-miR-142	CTGTCAGTCTGTCCTC	58332656	58332671	−	Chr17	Up

GC-sensitive miRNAs presenting positive GREs (pGRE).

## Abbreviations

GC	Glucocorticoid
IBD	Inflammatory bowel disease
miRNA	Micro RNA
GR	Glucocorticoid receptor
GRE	Glucocorticoid responsive elements
